# Georgia Medical Society, Savannah

**Published:** 1875-08

**Authors:** 


### Georgia Medical Society.

HENRY LeHARDY, M.D., Reporter.

SAVANNAH, GA., May 19, 1875.

SUBJECT OF HERPES.

Dr. Juriah Harriss is of the opinion that the disease is self-limited, and that remedies do little good. Gentle local applications are generally sufficient.

Dr. T. J. Charlton.—Some forms of herpes are very difficult to cure. Mentions cases in which herpes preputialis became a syphilitic chancre by contamination with a syphilitic subject.

Dr. Harriss believes that parties who are not married, and only have occasional sexual connection, are the most apt to contract herpes preputialis.

Dr. Thomas Smith believes that herpes preputialis occurs most frequently in persons having a long prepuce. Mentions circumcision as the best preventive.

Dr. W. G. Bulloch thinks that its differential diagnosis from exanthematous disease is very easy. In the treatment of herpes he generally uses lotions of acetate of lead, alum, etc., and when the system is debilitated uses tonics and alteratives. He believes that herpes preputialis is often connected with irritation of the urethra.

SAVANNAH, GA., June 2, 1875.

SUBJECT OF ERYSIPELAS.

Dr. W. M. Charters spoke of an epidemic of phlegmonous erysipelas which occurred in Ohio and Indiana thirty years ago; the cases involved the throat and neck principally, and were called "black tongue" by the inhabitants. The disease was very fatal. The treatment of erysipelas by preparations of iron was not known at that time, and all other remedies were unsuccessful. He mentions some thirty cases of that disease which occurred in Savannah in the beginning of 1851. These cases were remarkable for their severity, coupled with the small ratio of mortality. He treated them with the muriated tincture of iron, and when the disease attacked the face he used external applications of collodion with a little tincture of iodine, and palliatives. When the disease does not attack the throat it is seldom a very dangerous one. He believes that the tincture of iron is the best remedy.

Dr. J. C. LeHardy has met three forms of erysipelas in this city: the first affecting the epidermis and rete alone; the second the whole of the dermic tissues; and the third the dermis and areolar tissues beneath. In the first form the patient complains of a burning sensation in the skin; the epidermis and rete then become inflamed. It never spreads much, but is liable to metastasis, and is often intractable to remedies. The second form is slower in its progress, but more dangerous and more liable to affect the internal organs. The third form is still slower in its progress, and the inflammation passing on to suppuration, sometimes involves a large surface of the body, requiring large incisions for the relief of the patient.

Dr. J. C. Habersham mentions a case of erysipelas in which the temperature ran up to 112 a few hours before death occurred. The attack was preceded by one of bilious fever.

---

SAVANNAH, GA., June 9th, 1875.

SUBJECT OF HEMORRHOIDS.

Dr. J. C. LeHardy has derived more good from leeching than anything else in the treatment of hemorrhoids, excepting surgical operation. As soon as the conjestion is removed, he uses salves and soothing applications.

Dr. Rob't P. Myers has cured a case of internal bleeding hemorrhoids by means of injections of wine of ergot into the rectum.

Dr. W. M. Charters prefers the operation of crushing to any other, as it produces very little bleeding and very small scars. He has used in some cases with satisfaction a pair of bone nippers, of which the blades were lined with linen to blunt their cutting edges. Would like to hear of something that would procure speedy relief in cases of hemorrhoids occurring in pregnant women, producing a great deal of pain and annoyance. He has treated cases successfully by means of simple enemata of cold water, never allowing the bowels to act without a previous injection of cold water.

Dr. G. H. Stone has used the elastic ligature, together with the nitric acid, with great satisfaction. He renews the ligature daily.

Dr. Juriah Harriss thinks that external piles only require incisions, followed by soothing applications. If the patients desire to have the palliative treatment only, they should not be allowed to have any actions from the bowels during the day; they should empty the bowels at night, just before going to bed. He objects to the old plan of operating with the ligature; and when it is necessary to operate, he prefers the use of the ecraseur, or cauterization with nitric acid.

Dr. Theo. Starbuck speaks of a method of operating which consists of dissecting up the tumor until the vessel supplying it is found, when a ligature is applied upon it. Has derived much benefit from the use of injections of fiuid extract of ergot, with extract of opium and tannin.

Dr. Wm. H. Elliott has found the use of Smith's clamp to succeed very well except when the tumors are situated very high up in the rectum. He uses the actual cautery also.

Dr. T. J. Charlton has used the clamp, but prefers the old plan of ligating internal piles to any other treatment. He finds that the clamp cuts too much and often produces much bleeding. He uses the knife or scissors in the treatment of external hemorrhoids. He believes that the ligature effects the cure of internal hemorrhoids in the shortest time.

Dr. W. G. Bulloch thinks also that the old plan of ligating piles is the best. He would not use the clamp in very large piles.

